# Preoperative neutrophil-to-lymphocyte ratio plus platelet-to-lymphocyte ratio in predicting survival for patients with stage I–II gastric cancer

**DOI:** 10.1186/s40880-016-0122-2

**Published:** 2016-06-24

**Authors:** Xiaowei Sun, Xuechao Liu, Jianjun Liu, Shangxiang Chen, Dazhi Xu, Wei Li, Youqing Zhan, Yuanfang Li, Yingbo Chen, Zhiwei Zhou

**Affiliations:** Department of Gastric and Pancreatic Surgery, State Key Laboratory of Oncology in South China, Collaborative Innovation Center for Cancer Medicine, Sun Yat-sen University Cancer Center, 651 Dongfeng Road East, Guangzhou, 510060 Guangdong P.R. China

**Keywords:** Neutrophil-to-lymphocyte ratio, Platelet-to-lymphocyte ratio, Prognosis, Gastric cancer

## Abstract

**Background:**

The preoperative neutrophil-to-lymphocyte ratio (NLR) and the platelet-to-lymphocyte ratio (PLR) are associated with poor prognosis of gastric cancer. We aimed to determine whether the combination of NLR and PLR (NLR–PLR) could better predict survival of patients after curative resection for stage I–II gastric cancer.

**Methods:**

We collected data from the medical records of patients with stage I–II gastric cancer undergoing curative resection between December 2000 and November 2012 at the Sun Yat-sen Cancer Center. The preoperative NLR–PLR was calculated as follows: patients with both elevated NLR (≥2.1) and PLR (≥120) were given a score of 2, and patients with only one or neither were given a score of 1 or 0, respectively.

**Results:**

Kaplan–Meier analysis and log-rank tests revealed significant differences in overall survival (OS) among patients with NLR–PLR scores of 0, 1 and 2 (*P* < 0.001). Multivariate analysis showed that OS was independently associated with the NLR–PLR score [hazard ratio (HR) = 1.51, 95% confidence interval (CI) 1.02–2.24, *P* = 0.039] and TNM stage (HR = 1.36, 95% CI 1.01–1.83, *P* = 0.041). However, other systemic inflammation-based prognostic scores, including the modified Glasgow prognostic score, the prognostic nutritional index, and the combination of platelet count and NLR, were not. In TNM stage-stratified analysis, the prognostic significance of NLR–PLR was maintained in patients with stage I (*P* < 0.001) and stage II cancers (*P* = 0.022). In addition, the area under the receiver operating characteristic curve for the NLR–PLR score was higher than those of other systemic inflammation-based prognostic scores (*P* = 0.001).

**Conclusion:**

The preoperative NLR-PLR score is a useful predictor of postoperative survival in the patients with stage I–II gastric cancer and may help identify high-risk patients for rational therapy and timely follow-up.

## Background

Gastric cancer is the second leading cause of cancer-related death worldwide, with approximately 1 million new cases diagnosed annually [1, 2]. Despite advancements in surgical techniques and adjuvant chemotherapy, the postoperative overall survival (OS) of gastric cancer patients is still short, given the relatively late stage of disease at diagnosis [3, 4]. Identifying prognostic factors of OS may help identify patients at high risk for close follow-up and guide treatment for selected patients.

A systemic inflammatory response is important in carcinogenesis and tumor progression and is associated with short postoperative survival in patients with various types of cancer [5–8]. Several systemic inflammation-based scores such as the neutrophil-to-lymphocyte ratio (NLR), platelet-to-lymphocyte ratio (PLR), modified glasgow prognostic score (mGPS), prognostic nutritional index (PNI), and the combination of platelet count (PLT) and NLR (PLT–NLR) have prognostic values in many types of cancer, including gastric cancer [9–13]. A study from our center found that an elevated preoperative GPS was superior to NLR and PLR for predicting prognosis of the patients with stage III gastric cancer [14]. However, appropriate predictors for stage I–II gastric cancers remain unclear. The patients with stage I–II gastric cancer have a relatively good prognosis, but recurrence or metastasis is often difficult to predict in such patients. Therefore, a preoperative predictor of postoperative survival in the patients with stage I–II gastric cancer would be useful.

To test our hypothesis that an integrated indicator may better reflect the balance of host inflammation, we determined the prognostic value of NLR–PLR in predicting postoperative survival of the patients with stage I–II gastric cancer.

## Patients and methods

The study was approved by the Ethical Committee of Sun Yat-sen University Cancer Center and complied with the standards of the Declaration of Helsinki.

### Patient selection

Data were collected from the records of patients with gastric cancer undergoing curative resection (D2 lymphadenectomy) between December 2000 and November 2012 at the Sun Yat-sen University Cancer Center. The diagnosis of gastric cancer was confirmed pathologically with postoperative histological specimens, and clinical stage was determined as I or II according to the American Joint Committee on Cancer tumor-nodes-metastasis (TNM) staging system [15]. The patients who had inflammatory diseases for nearly 1 month and those who died of non-cancer-related causes were excluded from the study. The patients undergoing neoadjuvant chemotherapy or radiotherapy were also excluded. We collected data on age, sex, preoperative laboratory examinations, postoperative tumor characteristics, and survival duration. Blood samples taken within 1 week before surgery were tested for concentrations of C-reactive protein (CRP) and albumin and for lymphocyte, neutrophil, and platelet counts.

### Follow-up

After surgery, all patients were followed regularly every 6 months for the first 2 years and every year thereafter with laboratory tests and dynamic computed tomography (CT) and gastroscopy. The last follow-up date was July 8, 2014. Survival duration was measured from the date of surgery until death or last follow-up.

### Inflammation-based scores

The NLR was defined as the absolute neutrophil count divided by the absolute lymphocyte count. Similarly, the PLR was defined as the absolute platelet count divided by the absolute lymphocyte count.

The mGPS was calculated as previously described [11]: patients were given an mGPS score of 2 if they had a CRP concentration greater than 10 mg/L and an albumin concentration less than 35 g/L; an mGPS score of 1 if they had a CRP concentration greater than 10 mg/L and an albumin concentration of 35 g/L or higher; and an mGPS of 0 if they had a CRP concentration of 10 mg/L or less.

The PNI was calculated from the serum albumin concentration and absolute lymphocyte count: the patients in whom a combined albumin (g/L) × total lymphocyte count (×10^9^/L) was 45 or higher were assigned a PNI score of 0; the patients with a count less than 45 were assigned a score of 1 [12].

The PLT–NLR was assigned as follows: the patients with an elevated PLT (>300 × 10^9^/L) and an elevated NLR (>3) were assigned a score of 2; the patients with one abnormal value were assigned a score of 1; and the patients with no abnormal values were assigned a score of 0 [13].

The NLR–PLR was calculated as follows: the patients with both an elevated NLR (≥2.1) and PLR (≥120) were assigned a score of 2; the patients with only one elevated value were assigned a score of 1; the patients with no elevated values were assigned a score of 0.

### Statistical methods

Data are reported as means and 95% confidence intervals (CI). The relationships between the inflammation-based scores and the clinicopathologic characteristics were analyzed with the Pearson Chi square test. Correlation between NLR and PLR was assessed with Pearson’s correlation coefficient. Kaplan–Meier analysis and log-rank test were used to compare postoperative survival among groups with different NLR–PLR scores. Continuous variables meeting the assumption of linearity in the logit were categorized by the optimal cutoff value as determined by receiver operating characteristic (ROC) curve analyses.

Covariates significant in univariate analysis at the 0.05 level and not significantly associated with others were included in a Cox proportional hazard model for the final multivariate analysis. The prognostic value of the systemic inflammation-based prognostic scores was compared using the ROC analyses. Alpha was set at 0.05, and all data were analyzed with the SPSS 19.0 statistical software program (IBM Corporation, Armonk, NY, USA).

## Results

### Patient characteristics

Of the 305 patients enrolled, 168 (55.1%) were men, and 137 (44.9%) were women (Table 1). The median age was 57 years (range, 19–89 years). The median follow-up duration was 61 months (range, 1–162 months). Before 2005, CRP was not tested regularly in our center. Therefore, 90 patients were not tested for CRP, and we lacked associated data for mGPS. By July 8, 2014, 70 (23.0%) patients died, and 235 (77.0%) were still alive. The median OS was not approached. The 3- and 5-year OS rates were 84.9% and 79.7%. The NLR-PLR score was 0 for 120 patients, 1 for 96 patients, and 2 for 89 patients.

**Table 1:** Clinical and laboratory characteristics as well as overall survival of the 305 patients with stage I–II gastric cancer after resection

Characteristic	Patients [cases (%)]	Overall survival (months)^a^	*P* ^b^
Age			<0.001
<60 years	168 (55.1)	132.6 (123.7–141.5)	
≥60 years	137 (44.9)	110.5 (98.2–122.9)	
Sex			0.072
Male	202 (66.2)	115.7 (105.6–125.8)	
Female	103 (33.8)	135.0 (123.7–146.3)	
Tumor location in the stomach			<0.001
Upper third	87 (28.5)	82.0 (70.6–93.4)	
Middle third	59 (19.3)	131.7 (116.4–147.1)	
Lower third	159 (52.1)	133.3 (123.3–143.3)	
Tumor size			0.343
<3 cm	156 (51.1)	123.3 (112.8–133.9)	
≥3 cm	149 (48.9)	121.5 (110.4–132.6)	
TNM stage			0.001
IA	56 (18.4)	108.1 (102.4–113.8)	
IB	53 (17.4)	112.9 (100.5–125.3)	
IIA	50 (16.4)	115.9 (98.2–133.6)	
IIB	146 (47.9)	111.1 (99.0–123.2)	
mGPS^c^			<0.001
0	158 (51.8)	75.1 (70.5–79.6)	
1	44 (14.4)	67.4 (58.9–75.9)	
2	13 (4.3)	42.7 (25.8–59.6)	
PNI			0.010
0	282 (92.5)	126.7 (118.8–134.7)	
1	23 (7.5)	68.5 (49.7–87.4)	
PLT–NLR			0.009
0	210 (68.9)	128.3 (119.9–136.7)	
1	78 (25.6)	93.7 (79.5–107.8)	
2	17 (5.6)	125.3 (96.2–154.4)	
NLR–PLR			<0.001
0	120 (39.3)	142.2 (133.7–150.8)	
1	96 (31.5)	119.0 (105.5–132.5)	
2	89 (29.2)	100.9 (85.4–116.4)	

*mGPS* modified glasgow prognostic score, *PNI* prognostic nutritional index, *PLT* platelet count, *NLR* neutrophil-to-lymphocyte ratio, *PLR* platelet-to-lymphocyte ratio

^a^The median survival for most groups was not arrived because most patients were still alive at the last follow-up. Therefore, survival data are presented as mean with the 95% confidence interval (CI) in parentheses

^b^Kaplan–Meier survival analysis

^c^Before 2005, CRP was not tested regularly in our center. Therefore, CRP data are not available for 90 patients, and we lacked associated data for mGPS

### Selection of optimal cutoff values

**Fig. 1:**
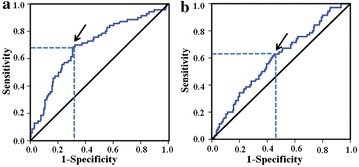
Receiver operating characteristic curve for the **a** neutrophil-to-lymphocyte ratio (NLR) and **b** platelet-to-lymphocyte ratio (PLR). *Arrows* indicate optimal cutoff values

According to the results of ROC analysis (Fig. 1), the patients were categorized into two groups by the optimal cutoff values of NLR (low, <2.1; high, ≥2.1) and PLR (low, <120; high, ≥120). Moreover, NLR was positively correlated with PLR (*r* = 0.37, *P* < 0.001).

### Survival and prognostic factors

**Fig. 2:**
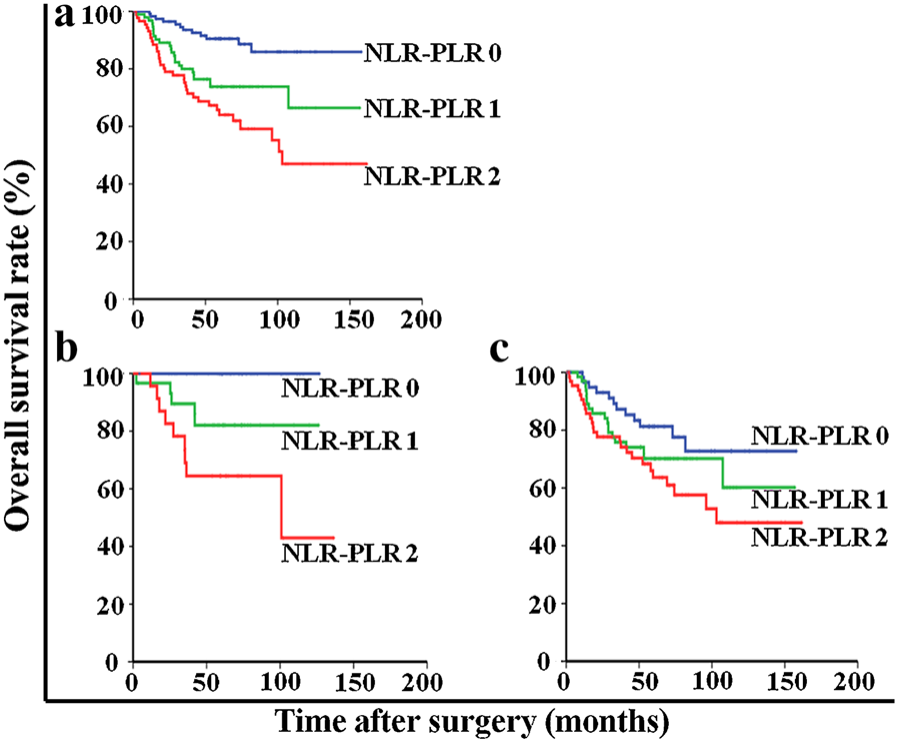
Kaplan–Meier overall survival curves for the patients with stage I–II gastric cancer after curative resection. **a** patients with either stage I or II gastric cancer. **b** only patients with stage I gastric cancer. **c** only patients with stage II gastric cancer. The survival curves show that the patients with an NLR–PLR score of 2 had significantly lower overall survival rates than the patients with an NLR–PLR score of 0 or 1. NLR–PLR, the combination of neutrophil-to-lymphocyte ratio and platelet-to-lymphocyte ratio

An NLR–PLR score of 2 was associated with older age (*P* = 0.026), tumor location in the upper third of the stomach (*P* = 0.029), larger tumor size (*P* < 0.001), higher TNM stage (*P* = 0.034), higher mGPS score (*P* < 0.001), higher PNI score (*P* < 0.001), and high PLT-NLR (*P* < 0.001; Table 2). The mean OS was significantly longer for the patients with a NLR–PLR score of 0 than for the patients with scores of 1 or 2 (142.2 vs. 119.0 and 110.9 months; *P* < 0.001). Of note, the mean OS was shorter in stage I patients with an NLR–PLR score of 2 (89.4 months) than in stage II patients with an NLR–PLR score of 0 (127.3 months), although the difference was not significant (*P* = 0.074). The 5-year OS rates of the patients with NLR-PLR scores of 0, 1, and 2 were 90.5%, 73.8%, and 64.0%, respectively (*P* < 0.001; Fig. 2a).

**Table 2:** Relationships between NLR–PLR and clinicopathologic characteristics of 305 patients with stage I–II gastric cancer

Characteristic	NLR–PLR			***P***
	0	1	2	
Total	120	96	89	
Age				0.026
<60 years	77	50	41	
≥60 years	43	46	48	
Sex				0.735
Male	81	65	56	
Female	39	31	33	
Tumor location in the stomach				0.029
Upper third	24	28	35	
Middle third	29	15	15	
Lower third	67	53	39	
Tumor size				<0.001
<3 cm	77	48	31	
≥3 cm	43	48	58	
TNM stage				0.034
IA	31	17	8	
IB	25	13	15	
IIA	16	18	16	
IIB	48	48	50	
mGPS				<0.001
0	68	57	33	
1	10	11	23	
2	2	4	7	
PNI				<0.001
0	120	94	68	
1	0	2	21	
NLR–PLR				<0.001
0	114	70	26	
1	6	26	46	
2	0	0	17	

*mGPS* modified glasgow prognostic score, *PNI* prognostic nutritional index, *PLT* platelet count, *NLR* neutrophil-to-lymphocyte ratio, *PLR* platelet-to-lymphocyte ratio

Univariate and multivariate analyses were performed to assess the relationship between clinical characteristics and OS (Table 3). Multivariate analyses showed that OS was independently associated with the NLR–PLR score (HR = 1.51, 95% CI 1.02–2.24, *P* = 0.039) and TNM stage (HR = 1.36, 95% CI 1.01–1.83, *P* = 0.041). When stratified by TNM stage, the prognostic value of NLR–PLR scores remained in the patients with stage I (*P* < 0.001; Fig. 2b) and stage II gastric cancer (*P* = 0.022; Fig. 2c).

**Table 3:** Univariate and multivariate Cox regression analyses of overall survival in 305 patients with stage I–II gastric cancer

Characteristic	Univariate analysis		Multivariate analysis	
	Hazard ratio (95% CI)	*P*	Hazard ratio (95% CI)	*P*
Age (<60 vs. ≥60 years)	2.43 (1.49–3.96)	<0.001	1.62 (0.91–2.89)	0.100
Sex (male vs. female)	0.61 (0.36–1.05)	0.075		
Tumor location in the stomach (upper vs. middle vs. lower third)	0.59 (0.45–0.77)	<0.001	0.77 (0.56–1.07)	0.119
Tumor size (<3 vs. ≥3 cm)	1.26 (0.78–2.01)	0.344		
TNM stage (IA vs. IB vs. IIA vs. IIB)	1.60 (1.25–2.05)	<0.001	1.36 (1.01–1.83)	0.041
mGPS (0 vs. 1 vs. 2)	1.86 (1.25–2.76)	0.002	1.24 (0.80–1.93)	0.345
PNI (0 vs. 1)	2.36 (1.21–4.62)	0.012	0.78 (0.31–1.93)	0.589
PLT–NLR (0 vs. 1 vs. 2)	1.40 (0.99–1.97)	0.057		
NLR–PLR (0 vs. 1 vs. 2)	2.018 (1.50-2.72)	<0.001	1.51 (1.02–2.24)	0.039

*mGPS* modified glasgow prognostic score, *PNI* prognostic nutritional index, *PLT* platelet count, *NLR* neutrophil-to-lymphocyte ratio, *PLR* platelet-to-lymphocyte ratio

### Comparison between inflammation-based scores

**Fig. 3:**
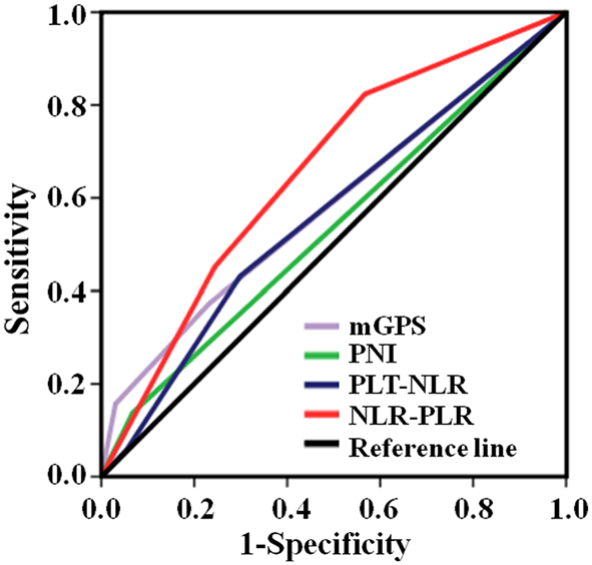
Areas under the receiver operating characteristic curves predicting survival among 305 patients with stage I–II gastric cancer after curative resection. *mGPS* modified Glasgow prognostic score, *PNI* prognostic nutritional index, *NLR–PLR* the combination of neutrophil-to-lymphocyte ratio and platelet-to-lymphocyte ratio

To further evaluate the prognostic values of the systemic inflammation-based prognostic scores, ROC analysis was performed, and AUC values were compared (Fig. 3). The NLR–PLR score had a higher AUC value (0.66; *P* = 0.001) than mGPS, PNI, and PLT–NLR (Table 4).

**Table 4:** Areas under the receiver operating characteristics (ROC) curve for four inflammation-based prognostic scores for predicting overall survival in 305 patients with stage I–II gastric cancer after resection

Prognostic score	Area under the ROC curve (95% CI)	*P*
mGPS	0.58 (0.49–0.68)	0.074
PNI	0.54 (0.44–0.63)	0.449
PLT–NLR	0.56 (0.47–0.66)	0.173
NLR–PLR	0.66 (0.57–0.74)	0.001

*mGPS* modified glasgow prognostic score, *PNI* prognostic nutritional index, *PLT* platelet count, *NLR* neutrophil-to-lymphocyte ratio, *PLR* platelet-to-lymphocyte ratio

## Discussion

Accumulating evidence indicates that a systemic inflammatory response is associated with a poor outcome in many types of cancer [16–19]. In our study, we determined the prognostic value of the NLR–PLR score in the patients with stage I–II gastric cancer. We found that the score independently predicted OS in these patients better than other inflammation-based prognostic scores we tested.

Cancer and inflammation are linked [20, 21]. However, the mechanisms by which inflammatory response induces a poor outcome are still ambiguous and poorly understood. Several mechanisms have been proposed. First, the tumor microenvironment inhabited by inflammatory cells is crucial in carcinogenesis, promoting tumor cell proliferation and migration [22, 23]. Second, tumor cells themselves and tumor-associated leukocytes could produce various inflammatory cytokines, such as tumor necrosis factor-alpha, interleukin-6, and vascular endothelial growth factor. These inflammatory cytokines and chemokines have powerful effects on cancer growth, invasion, and metastasis [24]. Third, cancer-related inflammation can recruit regulatory T cells and activate chemokines, which suppress antitumor immunity [25, 26].

The use of cellular components of a systemic inflammatory response for predicting survival has received increased attention. A recent meta-analysis of 10 studies (involving a total of 2952 patients) found that a high NLR might be associated with poor prognosis for patients with gastric cancer [27]. In addition, a high PLR appeared to be associated with short progression-free and overall survival in the patients with advanced gastric cancer treated with chemotherapy [28].

We found that a higher NLR–PLR score was associated with larger tumor size and more advanced TNM stage. This finding supported those of previous studies, in which inflammation-based prognostic scores significantly paralleled tumor progression [29–31]. In our study, Kaplan–Meier analysis showed that the NLR–PLR score was associated with OS (*P* < 0.001). The mean OS was shorter in stage I patients with a NLR-PLR score of 2 (89 months) than in stage II patients with a NLR–PLR score of 0 (127 months). Although this difference was not significant (*P* = 0.074), it was large enough to identify more patients at high risk undergoing curative resection than that afforded by TNM stage alone. In addition, multivariate analysis revealed that the NLR–PLR score was an independent predictor of OS. More importantly, the prognostic value of the NLR–PLR score was maintained in the patients with stage I (*P* < 0.001) and stage II cancers (*P* = 0.022).

We also found that several other established inflammation-based prognostic scores lacked independent prognostic significance in multivariate analyses, although they were significantly associated with OS in univariate analysis. Obviously, in the context of stage I–II gastric cancer, the NLR–PLR score exerted more potent prognostic value than did the mGPS, PNI, and PLT–NLR. The area under the ROC curve was larger for the NLR–PLR score than for other prognostic scores. These results strongly support the prognostic value of the NLR–PLR score in the context of stage I–II gastric cancer patients undergoing curative resection.

The major limitations of the present study were its retrospective nature and the single-center experience. In addition, we lack data on disease-free survival, although OS is considered the standard indicator of cancer prognosis [32]. However, our data were based on a large consecutive sample size and provided a valid basis to investigate the prognostic value of inflammatory markers. Furthermore, the surgical procedures (R0 resection plus D2 lymphadenectomy), laboratory tests, and patient follow-up were uniform during the entire study period.

## Conclusions

We found that the NLR–PLR score predicted OS better than did other established inflammation-based prognostic scores we tested in the patients with stage I–II gastric cancer. This score may help clinicians identify the patients at a high risk of recurrence for rational therapy and closer follow-up.
